# White Syndrome in *Acropora muricata:* Nonspecific bacterial infection and ciliate histophagy

**DOI:** 10.1111/mec.13097

**Published:** 2015-02-23

**Authors:** Michael Sweet, John Bythell

**Affiliations:** ^1^ Molecular Health and Disease LaboratoryCollege of Life and Natural SciencesUniversity of DerbyKedleston RoadDerbyDE56 0TAUK; ^2^ University of the South PacificLaucala CampusSuvaFiji

**Keywords:** antibiotics, disease, Philaster, Vibrio

## Abstract

Selective antibiotic treatment of white syndrome (WS)‐affected corals (*Acropora muricata*) from Fiji was used to identify 3 potential bacterial pathogens of the disease. Interestingly, the suite of bacterial associates of the disease was different to that recently identified using identical primer sets for WS on the GBR and in the Solomon Islands. In addition to the three bacterial pathogenic candidates and as previously shown for WS and more recently for white band disease (WBD) in the Caribbean, all samples of the disease were specifically associated with the histophagous ciliate *Philaster lucinda*. From the pattern of disease progression and histopathology in relation to the selective elimination of microbial groups, we conclude that these ‘white’ diseases are a result of a nonspecific bacterial infection and a ‘secondary’ infection by the *P. lucinda* ciliate. Although we have not observed the initiation of infection, a nonspecific, multispecies bacterial infection appears to be a corequirement for WS lesion progression and we hypothesize that the bacterial infection occurs initially, weakening the defences of the host to predation by the ciliates. Such ciliate histophagy gives rise to the characteristic white band of denuded coral skeleton that gives these diseases their names. The characteristics of the microbial communities of WBD and WS appear identical, and since the bacterial associates of WS vary geographically (and/or temporally), there appears to be no logical distinction between WS in the Indo‐Pacific and WBD in the Caribbean.

## Introduction

White plague (WP), white band disease (WBD) and other ‘white diseases’ of reef building corals (Bythell *et al*. 2004) cause significant coral mortality worldwide and have been responsible for epizootics that have had profound effects on coral community structure, particularly in areas of the Caribbean since the early 1970s (Aronson & Precht 2001; Miller *et al*. 2003). In the last 10–15 years, similar diseases have emerged in the Indo‐Pacific and are often collectively termed white syndrome (WS) (Willis *et al*. 2004). In the field, WS and similar white diseases are visibly characterized by an advancing full‐depth tissue lesion with a sharp demarcation between apparently normal tissues and the exposed coral exoskeleton (Willis *et al*. 2004). It is the exposed white skeleton that gives these diseases their names. Microscopically, there is no obvious tissue necrosis at the lesion boundary of WS (Ainsworth *et al*. 2007; Work & Aeby 2011), and despite numerous bacteria being proposed as the likely casual agents (Denner *et al*. 2003; Thompson *et al*. 2006; Sussman *et al*. 2008; Luna *et al*. 2010), no significant bacterial biomass has ever been observed (Ainsworth *et al*. 2007; Work & Aeby 2011; Sweet & Bythell 2012).

Recently, we also identified a number of ciliate species associated with disease lesions of both WS (Sweet & Bythell 2012) and WBD (Sweet *et al*. 2014). Several of these have been observed to ingest coral tissues at the lesion interface and are likely responsible for the macroscopic visible signs of the disease, namely the denuded coral skeleton adjacent to the lesion boundary and the absence of necrotic tissues. One of these ciliates in particular, *Philaster lucinda*, has been consistently observed in all cases of disease we have investigated to date, including *n* = 67 cases of WS and *n* = 36 WBD, in the Indo‐Pacific and the Caribbean, respectively. It is the only specific agent consistently observed in all cases of these diseases and also provides the only direct link to pathogenesis as it has been observed to ingest the coral tissues (Sweet & Bythell 2012). These studies have also shown a suite of 4–15 bacterial ribosomal types identified using culture‐independent 16S rRNA gene techniques that are associated with independent disease samples but absent from healthy samples. However, the suite of bacteria associated with these diseases appears to vary over both large geographical and temporal scales (Ben‐Haim & Rosenberg 2002; Denner *et al*. 2003; Barash *et al*. 2005; Gil‐Agudelo *et al*. 2006; Pantos & Bythell 2006; Luna *et al*. 2010; Sweet & Bythell 2012). Therefore in nature, the disease known as WS is associated with a number of different bacterial and ciliate associates as well as numerous other micro‐organisms such as fungi, viruses and other parasites (Work & Aeby 2011).

Because of the polymicrobial nature of WS and other white diseases, it is critical in challenge experiments to control for changes in the populations of other potential pathogens associated with these diseases, as treatment with the inoculum may cause physiological stress in the coral and alter competitive and host–pathogen interactions with other microbial associates. The *Philaster* sp. ciliate and several bacterial pathogens appear to be ubiquitous in coral reef environments, including aquarium systems (Sweet *et al*. 2013a). Therefore, in an attempt to address specific roles of these ubiquitous micro‐organisms, we used an alternative approach aimed to narrow down the identification of potential pathogens of WBD using experimental antibiotic treatments. This approach selectively inhibited bacterial and ciliate associates from diseased corals and allowed us to determine the effects on the disease progression and histopathology (Sweet *et al*. 2014). We found that by eliminating the entire suite of bacterial and ciliate associates of the disease, WBD progression could be halted. This finding is consistent with Kline & Vollmer (2011), who concluded that WBD is a bacterially mediated disease from transmission of the disease via a 0.45‐μm filtrate of diseased tissues that could be rendered ineffective by antibiotic treatment. However, at the time, interactions with ciliates and other potential pathogens already associated with the experimental samples were not investigated by Kline & Vollmer (2011). From the more recent study, we were able to conclude that the *Philaster* sp. ciliate appeared to not be the primary pathogen of WBD, as its elimination during certain treatments did not prevent lesion progression (Sweet *et al*. 2014). That said, the histopathology of the disease was altered upon reduction of populations of the ciliate, suggesting that it is a ‘secondary’ pathogen, contributing to the specific pathogenesis, rather than a secondary invader consuming tissues killed by a specific bacterial primary infection. Interestingly, during the same study, a suite of 3 bacterial ribotypes [*Vibrio charchariae* (KC737024), *Lactobacillus suebicus* (KC737026) and a *Bacillus* sp. (KC737032)] could not be eliminated as potential pathogens by the antibiotic treatments, indicating that one or a combination of more than one of these bacteria remain as the most likely primary casual agents of WBD. Yet ciliate histophagy is still required to produce the characteristic pathogenesis and visible disease signs.

In this study, a similar approach was used, employing three antibiotics targeting different bacterial and ciliate groups aimed at treating the coral *Acropora muricata* from Fiji displaying progressive WS lesions. We combined culture‐independent deep sequencing molecular techniques to characterize the bacterial and archaeal communities associated with the different treatments, denaturating gradient gel electrophoresis to characterize ciliate diversity, and histopathology as a tool to allow both detection of potential causative agents of the disease and the cellular response of the host. Our specific objectives were to describe the pathogenesis of *Acropora* white syndrome at the gross and cellular level and identify the potential causal agents.

## Materials and methods

*Acropora muricata* showing signs of white syndrome were collected from the Suva barrier reef, Fiji, and monitored for lesion progression. Only those displaying advancing lesions were used in the experiment. Coral nubbins (*n* = 24 × 5 cm^2^) showing signs of advancing WS were placed in recirculating natural seawater aquaria. Three replicate aquaria were used for each of the four treatments (giving *n* = 6 replicates per treatment). A further *n* = 6 healthy, nondiseased corals were placed in 3 aquaria as controls and maintained throughout the experiment. All corals were maintained in the aquarium for 72 h prior to the start of the experiment to allow for acclimatization and to monitor disease lesion progression. Three types of antibiotics were utilized in treatments to determine their effects on the diseased corals: ampicillin, metronidazole and paromomycin sulfate. A total of 100 μg/mL was used for all three antibiotics after preliminary laboratory trials on both bacteria and healthy corals. *N* = 6 corals with WS were left untreated in the tanks and sampled before all the tissue had been lost. Healthy corals were also treated with the antibiotics at the same dose rates to ensure that the antibiotic treatments did not have any adverse effect on the corals. All samples in these control treatments survived to the end of the experiment, with no visual appearance of tissue deterioration or discoloration.

Two treatments, ampicillin and metronidazole, were administered twice daily at 10:00 and 16:00 for 6 days until the end of the experiment. Paromomycin sulfate, in contrast, was applied only twice on the first day due to the cost of this antibiotic.

Sterile surgical gloves were worn at all times to avoid contamination. Samples were taken either when the corals had less than half their remaining tissue on the nubbins or at the end of the experiment. Samples were placed in Falcon tubes underwater and sealed. The water was then replaced with 100% ethanol for microbial analysis or preserved with 4% glutaraldehyde for histology.

### PCR and DGGE

Extraction, PCR and denaturing gradient gel electrophoresis were undertaken as described in Sweet & Bythell (2012). DNA was extracted from all samples using QIAGEN DNeasy Blood and Tissue kits, and bacterial 16S rRNA gene diversity was amplified using primers 357F and 518R (Sanchez *et al*. 2007). PCR protocols were as in Sweet & Bythell (2012). Ciliate 18S rRNA was amplified using primers CilF and CilDGGE‐r (Janse *et al*. 2004). PCR protocols were as in Sweet & Bythell (2012). For each of the above primer pairs, 30 μL PCR mixtures containing 1.5 mm MgCl_2_, 0.2 mm dNTP (promega), 0.5 mm of each primer, 2.5 Ul of Taq DNA polymerase (QBiogene), incubation buffer and 20 ng of template DNA were used as in Sweet & Bythell (2012). DGGE was performed as in Sweet & Bythell (2012) using the D‐Code universal mutation detection system (Bio‐Rad). PCR products were resolved on 10% (w/v) polyacrylamide gels for bacterial 16S rRNA gene diversity and 8% (w/v) for ciliate diversity. Bands of interest (those which explained the greatest differences/similarities between samples) were excised from DGGE gels, re‐amplified with the same original primers, labelled using Big Dye (Applied Biosystems) transformation sequence kit and sent to Genevision (Newcastle University, UK) for sequencing.

## 454 Sequencing and analysis

Only bacteria were assessed using 454 sequencing as ciliate communities showed low enough diversity to be represented with DGGE alone. Pyrosequencing PCR mixtures were the same as above with the substitution of HotStarTaq polymerase (Qiagen, Valencia, CA, USA). PCR conditions were the same as those in Sweet & Bythell (2012). Replicate PCR products (from the same sample) were used to reduce PCR bias. These were combined and cleaned using AMPure magnetic beads (Beckman Coulter Genomics, Danvers, MA, USA). Amplicon samples were quantified using the Qubit fluorometer (Invitrogen, Carlsbad, CA, USA) and pooled to an equimolar concentration. Sequences were run on 1/8th of a 454 FLX Titanium pico‐titre plate at Newgene in the Centre for Life, Newcastle, UK.

Sequences were filtered based on the following criteria: (i) sequences of <50 nucleotides, (ii) sequences containing ambiguous bases (Ns) and (iii) sequences containing primer mismatches. Any fitting into these criteria was discarded. Analysis using 2% single‐linkage preclustering (SLP) and average‐linkage clustering based on pairwise alignments (PW‐AL) were performed to remove sequencing based errors. The remaining sequences were denoised within QIIME (Caporaso *et al*. 2010). The resulting reads were checked for chimeras and clustered into 98% similarity operational taxonomic units (OTU) using the USEARCH algorithm in QIIME. All singletons (reads found only once in the whole data set) were excluded from further analyses. After blast searches on GenBank, we retained the best BLAST outputs, that is the most complete identifications, and compiled an OTU table, including all identified OTUs and respective read abundances. Effects of treatment were compared by visualizing differences using nonmetric multidimensional scaling (nMDS) based on the Bray–Curtis index of community dissimilarity (for both binary data and read abundance) (Oksanen *et al*. 2008). Analysis of similarity (ANOSIM) was also used to test for significant differences in OTU richness across the different treatments. ANOSIM assumptions of normality and homoscedasticity were tested. Statistical analyses on both OTU presence/absence and OTUs weighted by read abundance data showed the same trends, so we report only the latter. Read abundances across treatments and individual replicates were also used for assessing differences in selected taxonomic Class, Order, Family, Genus and where possible down to species level using one‐way ANOVA (raw OUT tables available on Dryad). All statistical tests and graphics were performed using PRIMER (Clarke & Gorley 2006). Sequence Read Archive under Accession no SRR7788726.

### Histology

Samples were collected as for microbial analysis; however, tissue samples were preserved with 4% glutaraldehyde for 24 h then stored in 100% EtOH until resin embedding in LR white (r). Survey sections of each tissue type were stained with the general DNA stain toluidine blue. Then samples were taken for transmission electron microscopy (TEM). TEM sections were dehydrated using 25% acetone, 50% acetone, 75% acetone, (30 min each) and 100% acetone (2 × 1 h). The samples were then impregnated with 25% LR White resin in acetone, 50% resin/acetone, 75% resin/acetone (1 h each), then 100% resin for a minimum of 3 changes over 24 h, with final embedding in 100% resin at 60°C for 24 h. Survey sections of 1 m were cut and stained with 1% toluidine blue in 1% Borax. Ultrathin sections (80 nm approx) were then cut using a diamond knife on a RMC MT‐XL ultramicrotome. These were then stretched with chloroform to eliminate compression and mounted on Pioloform filmed copper grids. Staining was with 2% aqueous uranyl acetate and lead citrate (Leica). The grids were then examined using a Philips CM 100 Compustage (FEI) Transmission Electron Microscope, and digital images were collected using an AMT CCD camera (Deben) at the Electron Microscopy Research Services Laboratory, Newcastle University.

## Results

**Figure 1:**
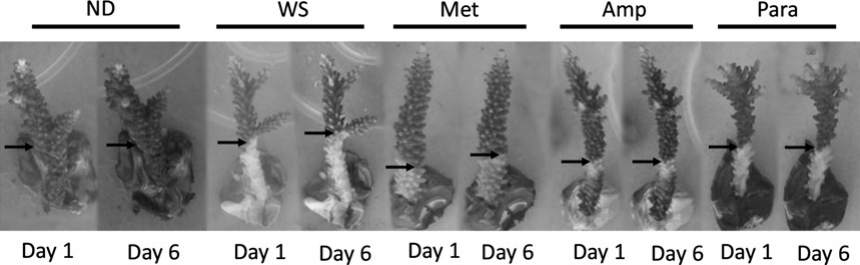
Representative photographs of *Acropora muricata* fragments used within the 6 day experiment. The image shows no visible changes in the nondiseased (ND) healthy corals, lesion advancement in the control untreated corals with white syndrome (WS), tissue advancement in the corals treated with metronidazole (Met) and no tissue advancement in the other two treatments, ampicillin (Amp) and paromomycin (Para).

Corals showing signs of WS were treated with three different antibiotics, ampicillin, metronidazole and paromomycin sulphate to assess the effect on their microbial consortium and the disease advance rate. Healthy, nondiseased (ND) corals were also initially treated with the antibiotics to assess the effect of the antibiotic on the health of the coral. All ND corals survived the 6 days of treatment and appeared normal (based on visual appearances) at the end of the experiment. A further set of ND corals (*n* = 6) was kept under the same aquarium conditions as the treated WS corals to determine any effect of housing in the tanks. All nondiseased corals survived and were visibly healthy at the end of the experiment. Conversely, all the diseased corals not treated with antibiotic continued to lose tissue throughout the duration of the experiment, and the advance rates of the disease lesion of these untreated corals (0.16 ± 0.001 cm/day) (Table 1) were within the range reported for WS in the field (Willis *et al*. 2004; Sweet & Bythell 2012). This indicates that the experimental conditions did not unduly affect disease progression or health of the corals. In specimens initially displaying an actively progressing WS lesion, two antibiotic treatments, ampicillin and paromomycin sulfate, completely arrested the advance of the lesion in all cases (Fig 1; *n* = 6 coral fragments per treatment). The rate of lesion progression in diseased corals treated with metronidazole slowed but continued to advance (Fig 1; Table 1).

**Table 1:** Tissue loss rates (cm^−3^/day) associated with healthy, nondiseased (ND) fragments of *Acropora muricata* under experimental condition, *A. muricata* showing signs of white syndrome (WS) and those treated with the three different antibiotics, ampicillin (Amp), paromomycin (Para) and metronidazole (Met)

Average Tissue Loss (cm^−3^/day)	ND	WS	Amp	Para	Met
Before Treatment	0	0.16 ± 0.001	0.17 ± 0.001	0.19 ± 0.001	0.16 ± 0.001
After Treatment	0	0.16 ± 0.001	0	0	0.08 ± 0.001

### Histology

**Figure 2:**
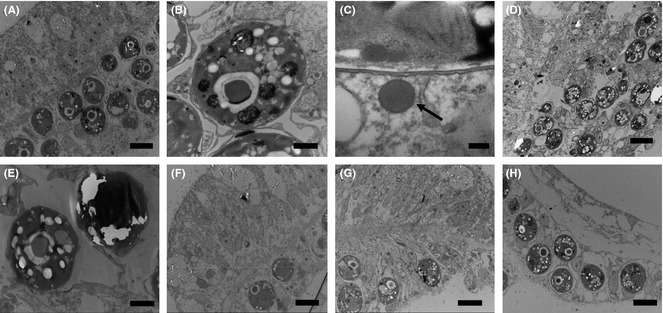
Transmission electron micrographs of the coral *Acropora muricata*; (A) nondiseased coral (Scale Bar = 10 μm); (B) symbiotic alga from a nondiseased sample (Scale Bar = 2 μm); (C) Virus like particle (arrow) associated with a symbiotic algal cell from a nondiseased coral (Scale Bar = 100 nm). (D) tissue from the WS lesion boundary (Scale Bar = 10 μm) and (E) symbiotic alga of a WS‐affected coral (Scale Bar = 2 μm). (F) tissue at the edge of a lesion arrested by treatment with ampicillin (Scale Bar 10 μm) and (G) tissue at the edge of a lesion arrested by treatment with paromomycin sulphate (Scale Bar 10 μm). (H) tissue at the lesion boundary of a WS‐affected coral treated with metronidazole (Scale Bar 10 μm). All tissues D‐H were sampled from within 2 mm of the lesion boundary.

**Figure 3:**
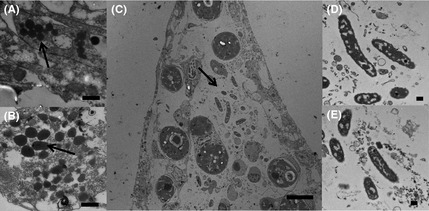
Transmission electron micrographs of the coral *Acropora muricata*; A and B shows possible bacterial associates (arrows) seen in all the tissues in all treatments: (A) an example of healthy tissues and (B) an example of WS‐affected samples (Scale Bar = 500 nm). (C) Illustrates the tissues of WS‐affected corals treated with metronidazole, showing the presence of a different type of rod‐shaped bacteria associated with disrupted tissues (Scale Bar 10 μm). These particular bacteria were only observed to be associated with metronidazole‐treated WS samples (D & E; Scale Bar 500 nm).

**Figure 4:**
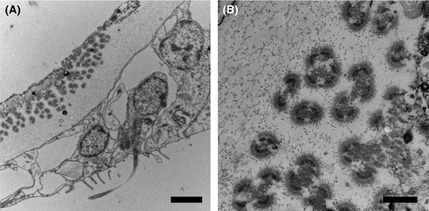
Transmission electron micrographs of virus like particles associated with white syndrome tissues treated with metronidazole (A; Scale Bar = 2 μm and B; Scale Bar = 500 nm).

Transmission electron microscopy of nondiseased corals used in the experiment showed that tissue structure was intact and appeared healthy (Fig 2A). The symbiotic algae showed no signs of stress‐related pathology (Fig 2B). However, circular virus like particles (VLPs), with a capsid diameter of 150 nm, were often associated with the outside edge of the symbiotic algae (Fig 2C). These particular VLPs occurred throughout all sample types at an average abundance of 71.4 cm^−2^ ± 10 (SD), and there was no significant difference between the abundance of these particular circular VLPs and treatment type (ANOSIM, *R* = 0.67, *P* = 0.67). The TEMs of corals showing signs of WS in contrast showed some evidence of tissue disruption and greater vacuolization of the symbiotic algae (Fig 2D, E). Tissue structure in the treated corals varied. Those corals treated with ampicillin and paromomycin sulfate showed similar structure to that of the healthy tissues (Fig 2F and 2G, respectively), while those treated with metronidazole showed disruption of the epidermal tissues, however, the *Symbiodinium* again appeared healthy (Fig 2H). Possible bacterial aggregates were associated with all samples (representatives seen in both healthy and WS‐affected tissues Fig 3A and B, respectively). Only in those treated with metronidazole were rod‐shaped bacteria observed, associated with the disrupted tissues (Fig 3C, D and E). Furthermore, morphologically different virus like particles (VLPs), than those associated with healthy tissues, were associated with metronidazole‐treated tissues only (Fig 4A, B). These VLPs showed a complex structure (Fig 4B), were very large in size (400–500 nm), were densely packed [214.28/cm^2^ ± 23 (SD)] and appeared localized at the site of the lesion interface and areas of fragmenting tissue.

### Bacterial 16S rRNA gene diversity

**Figure 5:**
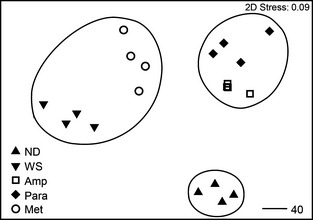
Multidimensional scaling (MDS) plot showing changes in 16S rRNA gene bacterial communities associated with nondiseased (ND) healthy corals, lesion advancement in the control untreated corals with white syndrome (WS), tissue advancement in the corals treated with metronidazole (Met) and no tissue advancement in the other two treatments, ampicillin (Amp) and paromomycin (Para). Contours show 40% similarities between replicates and different sample types.

There was a significant difference in 16S rRNA gene bacterial diversity in both the 454 sequencing and the DGGE (ANOSIM *R* = 0.998, *P* = 0.001 and ANOSIM *R* = 0.87, *P* = 0.023, respectively) between healthy and diseased corals (Table S1, Supporting information). There was also a significant difference between the healthy, diseased corals and all the treatments (ANOSIM *R* > 0.99, *P* < 0.001). Despite metronidazole targeting protozoans, it had a significant effect on the bacterial community, although the diversity was more similar (46%) to that associated with the nontreated corals exhibiting WS (Fig 5). Fifteen bacterial ribotypes were identified in this study as potential pathogens of WS, being consistently present in all samples of corals displaying WS but absent in healthy samples (Table S1, Supporting information). These were ribotypes related to *Leeuwenhoekiella*,* Tenacibaculum*,* Amoebophilaceae*,* Flammeovirgaceae*,* Aureispira*,* Stramenopiles*,* Lentisphaerales*,* Kordiimonadaceae*,* Helicobacteraceae*,* Alteromonadaceae*,* Glaciecola*,* Reinekea*,* Piscirickettsiaceae*,* Vibrio* and *Verrucomicrobiaceae species*. The *Vibrio* ribotype had 100% sequence similarity to *Vibrio tubiashii*.

The antibiotic treatments identified 12 of these potential pathogens as likely secondary invaders. These were still present in either the ampicillin and/or paromomycin sulfate treatments that arrested the lesion progression (*Tenacibaculum, Aureispira*,* Stramenopiles*,* Lentisphaerales*,* Kordiimonadaceae*,* Glaciecola* and the *Piscirickettsiaceae*) or undetectable in the metronidazole treatment in which the lesion continued to progress (*Alteromonadaceae*,* Helicobacteraceae*,* Amoebophilaceae*,* Flammeovirgaceae* and *Leeuwenhoekiella*).

Specifically only three bacterial ribotypes were absent in healthy tissues, present in diseased WS tissues, absent in both ampicillin and paromomycin sulfate treatments and still present in metronidazole treatments making them candidates for pathogenesis in WS in this instance. These included the *Reinekea*, the *Vibrio* and the *Verrucomicrobiaceae* ribosomal types.

### Ciliate 18S rRNA gene diversity

There was a diverse community of ciliates associated with WS samples, but ciliates were not detected in any of the healthy samples (Table 2). Ciliate ribotypes detected in samples with WS were related to: a *Diophrys*, a *Tiarinafusa*, two *Holosticha* species, a *Varistrombidium*, three uncultured alveolates, two *Protocruzia* species, two *Trochilioide* species, two *Uronema* species, *Philaster lucinda*, a *Glauconema* and a *Litonotus* species. Only the *Varistrombidium* and *P. lucinda* have previously been shown to be histophagous, ingesting the coral's symbiotic algae and burrowing underneath the apparently healthy tissue in advance of the disease lesion (Sweet & Bythell 2012). *Philaster lucinda* shared 100% sequence similarity of 596 base pairs to the recently described ciliate (Morph 1) associated with WS in both the Great Barrier Reef and the Solomon Islands (Sweet & Bythell 2012). This species was absent in all four samples treated with metronidazole, even though the lesion continued to progress in those treatments. However, the advance rate of the lesion slowed from an average of 0.16 to 0.08 cm^−3^ per day. Furthermore, the exposed skeleton on corals treated with metronidazole was discoloured and the lesion boundary did not exhibit the sharp demarcation, as is characteristic of this disease (Sweet & Bythell 2012). This result suggested a different pathology occurred with the absence of this ciliate species. Furthermore, histopathology showed significant disruption of the epidermis in this treatment. Finally, the *Varistrombidium* species, which is also histophagous, was present in ampicillin‐treated WS samples, even though this treatment effectively stopped the progression of the lesion, suggesting that the *Varistrombidium* is a secondary invader and not involved in pathogenesis.

**Table 2:**
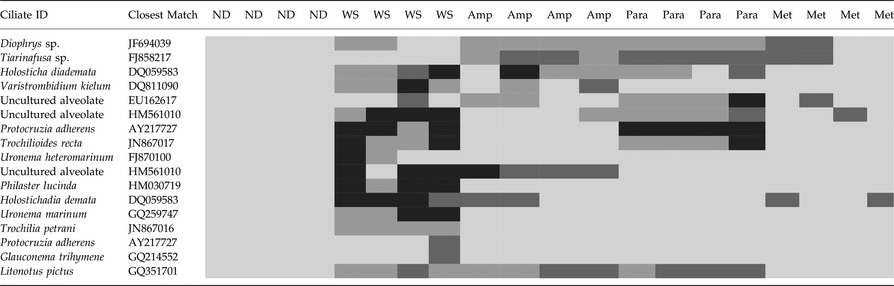
Heatmap showing the 18S rRNA gene ciliate diversity associated with healthy, nondiseased fragments of *Acropora muricata* under experimental condition, *A. muricata* showing signs of white syndrome (WS) and those treated with the three different antibiotics, ampicillin (Amp), paromomycin (Para) and metronidazole (Met)

## Discussion

Selective elimination of candidate pathogens using a variety of antibiotics in controlled experiments can aid in the identification of specific roles of these organisms with respect to disease causation. This study shows that the specific form(s) of WS sampled in this study were the result of a polymicrobial disease associated with a number of potential pathogens (15 bacteria and 7 ciliates identified in this study) that were consistently associated with all samples of disease and absent from healthy samples. This result supports previous studies on WS (Sussman *et al*. 2008; Luna *et al*. 2010; Sweet & Bythell 2012) as well as others such as white plague (Denner *et al*. 2003; Pantos *et al*. 2003; Bythell *et al*. 2004; Barash *et al*. 2005; Efrony *et al*. 2009; Atad *et al*. 2012) and black band disease (Cooney *et al*. 2002; Frias‐Lopez *et al*. 2003) which show multiple specific associates of any given diseased state. The combined elimination of three bacteria and one ciliate ribosomal type from this group of potential pathogens resulted in immediate and complete cessation of disease lesion progression, strongly indicating that one or a consortium of more than one (possibly all 4) of these candidate pathogens can be considered the ‘primary’ pathogen of WS in this instance. Only one of these candidate pathogens has been implicated in WS before, the ciliate *Philaster lucinda* (Sweet & Bythell 2012), which remains the only specific pathogen associated with the disease in all cases studied to date. However, as we have recently concluded for WBD in Caribbean acroporas, *Philaster lucinda* is more likely to be a ‘secondary’ pathogen contributing to pathogenesis and not a primary pathogen as the lesion continued to progress even though the ciliate was reduced to undetectable levels in the metronidazole treatment.

To date, the histophagous ciliate, *Philaster lucinda,* has not been detected in association with healthy corals in the natural environment, yet has been shown to be consistently present in the neighbouring reef environment, particularly associated with numerous marine algal species (Sweet *et al*. 2013b). It is therefore possible that they remain in these nearby reservoirs until the coral becomes compromised. With this in mind and the fact that two of the antibiotics utilized caused the lesion progression to cessate, the primary pathogen, at least in this instance, appears to be one or a combination of up to three of the bacterial agents. In the absence of the histophagous ciliate, these bacteria cause a degree of tissue necrosis that promotes lesion progression. In the natural disease state, these compromised tissues are apparently consumed by ciliates before necrosis occurs. However, without fine‐scale temporal sampling at the initiation of infection, it cannot be determined whether prior physical disruption by ciliates, or some other primary agent, may allow these specific bacterial agents to infect the tissues, and they may actually still be secondary agents, in a complicated disease process.

In this study, transmission electron microscopy showed the appearance of stress associated with the coral's symbiotic algae in progressive lesions but not in healthy tissues or those treatments which successfully stopped the disease. The symbiotic algae are a vital part of the coral holobiont and dysfunctional or damaged symbionts appear to be part of the aetiology of this disease. Damage to the symbionts may represent an additional stress to the coral and potentially provide a vicious cycle, further reducing host defences. This result, in which the algae are shown to be affected by the disease, is again similar to our recent study on WBD in the Caribbean acroporids.

The general absence of a significant population of bacteria and a lack of bacterial‐induced necrosis at the disease lesion interface of WS and similar ‘white diseases’ (Ainsworth *et al*. 2007; Work & Aeby 2011; Sweet & Bythell 2012) have been paradoxical to the finding of multiple bacterial causal agents for the disease by challenge experiments and culture‐independent studies (Ben‐Haim & Rosenberg 2002; Denner *et al*. 2003; Barash *et al*. 2005; Gil‐Agudelo *et al*. 2006; Pantos & Bythell 2006; Luna *et al*. 2010; Kline & Vollmer 2011; Sweet & Bythell 2012). This finding was also reflected in the present study. The altered histopathology that occurred in the metronidazole treatment may be explained by the lack of ciliates associated with this treatment, which would otherwise have consumed the tissues before necrosis could be detected. In the metronidazole‐treated corals, we were also able to locate numerous rod‐shaped bacteria associated with these fragmented tissues. This may explain why previous studies have failed to find bacteria associated with WS lesions, as infected tissues are rapidly consumed by the ciliates.

One further finding of note is the presence of *Bacteriovorax* in healthy tissues but absent in diseased samples. *Bacteriovorax* are predatory bacteria whose presence in host organisms is thought to shape the natural microbial community through trophic interactions (Thurber *et al*. 2009; Chen *et al*. 2012). Particularly, *Bacteriovorax* are known to prey on Gram‐negative bacteria such as members of the genus *Vibrio*. Its dynamics may therefore be of importance in maintaining a healthy population of microbes, and its subsequent absence in diseased tissues may then allow for pathogenic bacteria to infect (Chen *et al*. 2012).

Although detection of viruses was not a focus of this study, two main types were observed to be associated with the samples. One, circular, electron dense cored capsid VLP was consistently found in all sample types and appeared to be associated with the coral's symbiotic algae. Similar VLPs have previously been reported in *Acropora hyacinthus* (Pollock *et al*. 2014) and found in both healthy and diseased tissues. In contrast, the occurrence of a unique, larger VLP only present in metronidazole‐treated samples warrants further investigation. While this result is not sufficient to imply a role in disease causation, an increasing number of studies are showing the presence of viruses associated with disease lesions (Lohr *et al*. 2007; Lawrence *et al*. 2014a,b; Pollock *et al*. 2014; Yvan *et al*. 2014) and their role as a potential pathogen requires further study. However, interestingly, the study by Lawrence *et al*. (2014b) also highlighted the presence of ciliates within the diseased tissues of corals showing signs of *Porites* tissue loss (PorTL), a disease that fits within the generic description of WS disease.

Significant questions remain over the similarity between WS in the Indo‐Pacific and WBD in the Caribbean and between geographically and temporally separated WS cases. All these diseases are associated with the specific ciliate *Philaster lucinda,* and it remains the only agent with direct evidence for involvement in pathogenesis as it has been observed to consume intact coral tissues at the lesion interface (Sweet & Bythell 2012). However, we can conclude that the ciliate is a secondary pathogen due to its responses to antibiotic treatment. The diseases are also associated with a specific bacterial consortium that is absent in healthy coral and present in every case of the disease observed at any particular site. Thus, there is no distinction between WBD in the Caribbean and the equally variable geographical presentation of WS in the Pacific, either in associated microbial communities, histopathology or visible disease signs. The question then remains as to whether the different white syndromes and WBD consist of separate types of disease or are a single disease. It is possible that they are clearly separate diseases, caused by specific bacteria, but that the secondary, specific ciliate pathogen and other nonspecific pathogens cause disease signs that are indistinguishable. The fact that none of the specific agents associated with WS in Fiji in this study were previously detected in corals showing signs of WS in the GBR or Solomon Islands (Sweet & Bythell 2012) might suggest that they are separate diseases associated with specific pathogens. However, it is also possible that the bacterial infections are nonspecific and opportunistic (Lesser *et al*. 2007) and that any of a wide range of potential pathogens can act to cause initial infection and weaken host defences to allow the ciliate pathogens to invade. However, the fact that the bacterial consortium associated with the disease is so consistent between cases (sometimes separated by several km) and that none of these agents can be detected in healthy coral, even with deep sequencing, suggests that WS is not a completely nonspecific infection. Whatever the case, these diseases are certainly polymicrobial diseases, not only with bacteria and ciliates playing different roles in pathogenesis, but with multiple, specific bacterial associates consistently associated with the disease at a particular location and time. Further work is needed to determine whether this is simply a result of the ‘beta diversity’ associated with coral reef environments (whatever specific bacterial pathogens are available in the environment at any one time cause the disease) or whether specific disease consortia are associated with a number of specific diseases. It is also possible that the disease consortia are continually changing, possibly in response to changes in the host defences. This latter hypothesis differs from the hologenome theory of coral disease evolution (Rosenberg *et al*. 2007) in that changes in microbial consortia rather than individual pathogen selection and evolution would be required to enable coral diseases to persist in the environment.

## Conflict of interests

There are no conflict of interests associated with the manuscript.

M.S. designed and conducted the experiment, completed the laboratory work, analyzed the data and wrote the paper. J.B. wrote the paper.

## Data accessibility

All data necessary are included in the manuscript or available on GenBank Sequence Read Archive under Accession no SRR7788726. Raw OTU data have been submitted on Dryad doi:10.5061/dryad.jk34r.

## Supporting information


**Table S1.** Heatmap illustrating the clone libraries showing the 16S rRNA gene bacterial diversity associated with healthy, non‐diseased (ND) fragments of *Acropora muricata* under experimental condition, *A. muricata* showing signs of white syndrome (WS) and those treated with the three different antibiotics, Ampicillin (Amp), Paromomycin sulphate (Para) and Metronidazole (Met).
